# A split-GFP tool reveals differences in the sub-mitochondrial distribution of wt and mutant alpha-synuclein

**DOI:** 10.1038/s41419-019-2092-1

**Published:** 2019-11-12

**Authors:** Mattia Vicario, Domenico Cieri, Francesca Vallese, Cristina Catoni, Lucia Barazzuol, Paola Berto, Alessandro Grinzato, Laura Barbieri, Marisa Brini, Tito Calì

**Affiliations:** 1 Department of Biomedical Sciences, University of Padova, Padova, Italy; 2 Department of Biology, University of Padova, Padova, Italy; 3 Padova Neuroscience Center (PNC), University of Padova, Padova, Italy

**Keywords:** Cell signalling, Molecular neuroscience

## Abstract

Parkinson’s disease (PD), the second most common neurodegenerative disorder, is characterized by dopaminergic neuronal loss that initiates in the substantia nigra pars compacta and by the formation of intracellular inclusions mainly constituted by aberrant α-synuclein (α-syn) deposits known as Lewy bodies. Most cases of PD are sporadic, but about 10% are familial, among them those caused by mutations in *SNCA* gene have an autosomal dominant transmission. *SNCA* encodes α-syn, a small 140-amino acids protein that, under physiological conditions, is mainly localized at the presynaptic terminals. It is prevalently cytosolic, but its presence has been reported in the nucleus, in the mitochondria and, more recently, in the mitochondria-associated ER membranes (MAMs). Whether different cellular localizations may reflect specific α-syn activities is presently unclear and its action at mitochondrial level is still a matter of debate. Mounting evidence supports a role for α-syn in several mitochondria-derived activities, among which maintenance of mitochondrial morphology and modulation of complex I and ATP synthase activity. α-syn has been proposed to localize at the outer membrane (OMM), in the intermembrane space (IMS), at the inner membrane (IMM) and in the mitochondrial matrix, but a clear and comparative analysis of the sub-mitochondrial localization of WT and mutant α-syn is missing. Furthermore, the reasons for this spread sub-mitochondrial localization under physiological and pathological circumstances remain elusive. In this context, we decided to selectively monitor the sub-mitochondrial distribution of the WT and PD-related α-syn mutants A53T and A30P by taking advantage from a bimolecular fluorescence complementation (BiFC) approach. We also investigated whether cell stress could trigger α-syn translocation within the different mitochondrial sub-compartments and whether PD-related mutations could impinge on it. Interestingly, the artificial targeting of α-syn WT (but not of the mutants) to the mitochondrial matrix impacts on ATP production, suggesting a potential role within this compartment.

## Introduction

Parkinson’s disease (PD) affects 6 million individuals worldwide. The neuronal loss in the substantia nigra pars compacta^1^ and the formation of intracellular inclusions of aberrant α-synuclein (α-syn)^2^, whose autosomal dominant mutations^3^ are found in familial forms of the disease, are the main hallmarks. Mounting evidence indicates that α-syn regulates vesicles release at the synaptic level and stabilizes the assembly of *SNARE* complex^4–7^. Although prevalently cytosolic, α-syn can also be found in the nucleus^8–11^, in the mitochondria^12–17^ and in the mitochondria-associated ER membranes (MAMs) fraction^18,19^. Its close relationship with mitochondria has been extensively supported by convincing works showing altered mitochondrial functions and dynamics in different cellular and animal models where the expression level of α-syn was manipulated by overexpression and/or silencing and where α-syn mutants were introduced. Accumulation of WT α-syn causes a reduction in mitochondrial complex I activity^14,20–22^ while α-syn null mice display striking resistance to the neurotoxin 1-methyl-4-phenyl-1,2,3,6-tetrahydropyridine (MPTP)-induced degeneration of dopaminergic neurons and reduced dopamine release^23,24^. Alterations including increased oxidative stress, lipid abnormalities, complex I deficiency, increased mitochondrial fragmentation, loss of membrane potential and cytochrome c release were reported in mutant α-syn transgenic^25,26^ and null mice^27^, as well as in cells overexpressing wt α-syn^28^. Moreover, α-syn has been shown to participate in the maintenance of mitochondrial integrity by regulating the fission/fusion machinery and the autophagic process^18,29–31^. Finally, we have previously demonstrated that α-syn positively enhanced mitochondrial Ca^2+^ transients generated upon Ca^2+^ release from the endoplasmic reticulum (ER) by increasing the ER-mitochondria contact sites^32^. A dose-dependent mechanism of this action has been proposed by us^32^ and, more recently, confirmed to be important also for α-syn modulation of other mitochondria related activities^33–35^.

Interestingly, α-syn was found to localize both in vitro and in vivo at the outer membrane (OMM), the intermembrane space (IMS), the inner membrane or in the mitochondrial matrix depending on cell lines, species and culture conditions^12,13,15,19,36–39^. Whether the presence of α-syn at specific sub-mitochondrial localization could be related to precise physiological and pathological circumstances remains elusive. Thus, we decided to investigate the sub-mitochondrial distribution of the WT and the PD-associated mutants of α-syn. We also evaluated conditions that may favour α-syn translocation into mitochondria in order to identify possible peculiar function for the specific sub-organelle targeted α-syn.

We have applied a bimolecular fluorescence complementation (BiFC) approach^40–42^, previously developed^43^ and recently improved^44^ by our group, to selectively monitor the sub-mitochondrial distribution of WT and PD-related α-syn mutants A53T and A30P and test whether selected cellular stimuli could change their distribution.

This approach led us to identify WT and mutants α-syn pools that under basal conditions constitutively reside at the OMM and in the IMS. No α-syn molecules were instead detected in the mitochondrial matrix. Interestingly, a quantitative evaluation of the reconstituted fluorescent signal has permitted to establish that the presence of PD-related mutations A30P and A53T significantly enhanced the fraction of α-syn found at the IMS. Moreover, we have found that oxidative stress induction, complex I inhibition and impairment of the endosome-lysosome acidification system selectively promoted the accumulation of WT but not of A30P and A53T mutant α-syn within the IMS. Finally, we took advantage from the possibility to artificially targeting α-syn to the mitochondrial matrix and to monitor whether its presence inside this sub-mitochondrial compartment could affect bioenergetic metabolism. Intriguingly, we have found that the presence of WT α-syn in the mitochondrial matrix, but not that of the PD-related A30P and A53T mutants, was able to sustain mitochondrial ATP synthesis, underling a new possible physiological role for WT α-syn and a new pathological mechanism for PD-associated mutations.

## Results

### A split-GFP based tool to monitor sub-mitochondrial localization

**Fig. 1:**
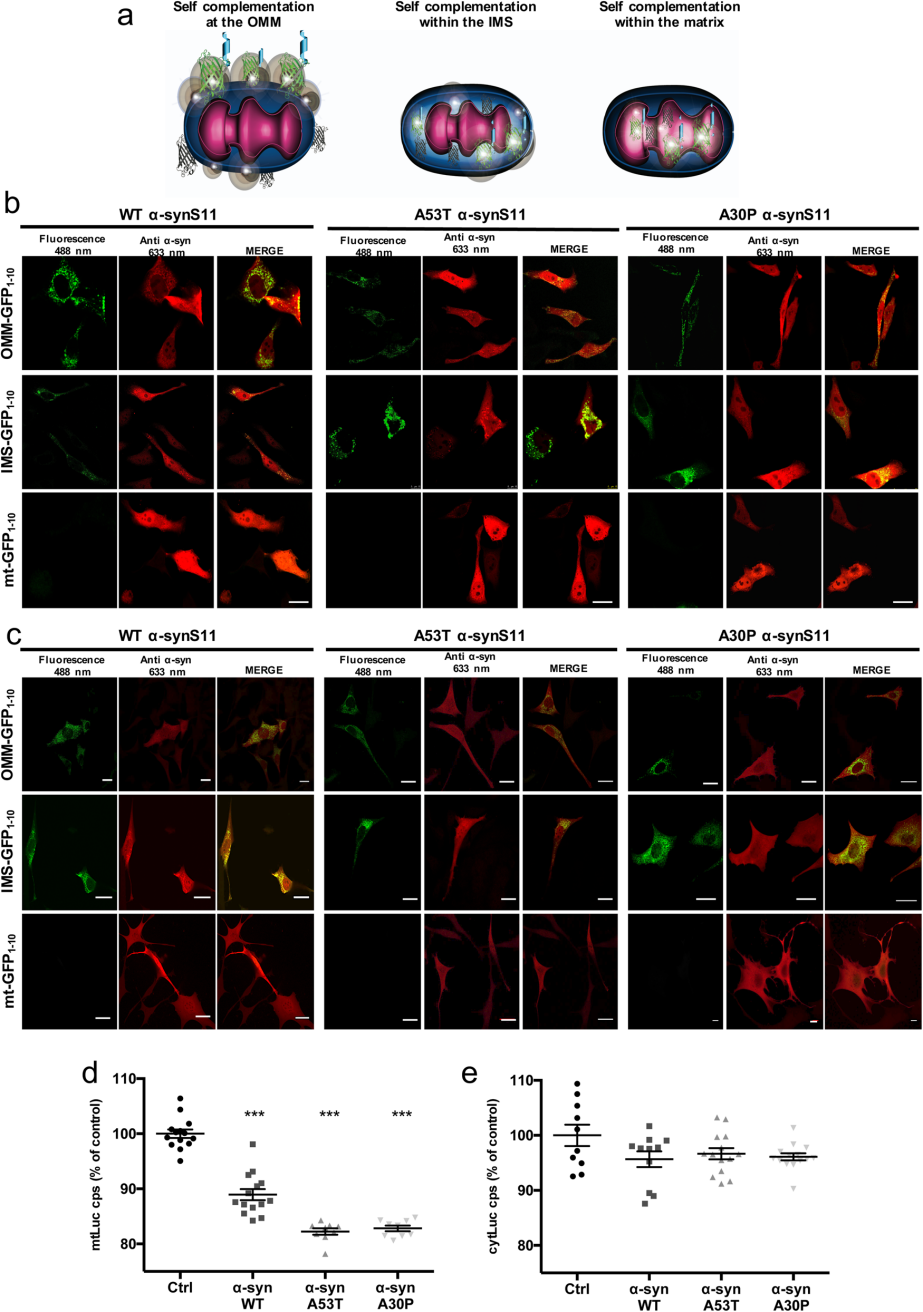
**a** Cartoon of targeted split-GFP chimeras are able to complement at the OMM, within the IMS and in the mitochondrial matrix. Sub-mitochondrial localization of wild type and mutant α-syn was analyzed in HeLa **b** and SHSY5Y **c** cells by the co-expression of the OMM, IMS and mitochondrial matrix targeted GFP_1-10_ non-fluorescent moiety and of the WT, A53T and A30P α-synS11. Confocal images were acquired at 488 and 594 nm excitation wavelength. Transfected cells were incubated with an anti α-syn primary antibody and stained with an Alexa 633 conjugated secondary antibody. Complementation of the GFP probes was revealed by fluorescent acquisition at 488 nm excitation wavelength. **d** Mitochondrial (****p < 0.0001 vs. control cells) and **e** cytosolic ATP production upon histamine stimulation measured by mtLuc probe in HeLa cells overexpressing α-syn wt and mutants. At least 9 independent measurements for three independent transfections have been done for each construct. One-way ANOVA retrieved a *p* value of 0.0001. Dunnett’s multiple comparison test retrieved a statistically significant difference between Ctrl and WT, A53T, and A30P *p* < 0.0001 **d** while no statistically significant differences were found for panel **e** with one-way ANOVA. Average values shown as mean % calculated from the counts per second (cps) with respect to control cells. Scale bar is 20 μm

In order to follow the exact sub-mitochondrial localisation of α-syn we applied the split-GFP based tool we had previously developed and described for other proteins of interest^43,44^. The GFP_1-10_ moiety lacking the S11 β-strand fused to the first 33 amino acids of the TOM20 N-terminal tail (OMM GFP_1–10_) or to the leader sequence of the inter membrane space protein LACTB (IMS GFP_1-10_) were employed to reveal the distribution of α-syn at the cytosolic surface of the outer mitochondrial membrane and at the inter membrane space, respectively^45,46^. To reveal the presence of α-syn in the mitochondrial matrix the GFP_1–10_ moiety was delivered to this sub-mitochondrial compartment by the fusion with the presequence of the subunit VIII of human cytochrome c oxidase (mt GFP_1–10_) as previously described^43^. As shown in Fig. 1a, these targeted GFP_1–10_ chimerae will properly reconstitute their fluorescence only when a protein tagged with the lacking S11 β-strand is located at the same compartment^43,44^. Control experiments confirmed the proper targeting and the absence of fluorescent signal of the mtGFP_1–10_ moiety, Fig. S1, as well as their ability to undergo self-complementation, Fig. S2^43,44^.

### WT and mutant α-syn reside at the OMM and IMS but not in the mitochondrial matrix and their overexpression modulates mitochondrial ATP production

**Fig. 2:**
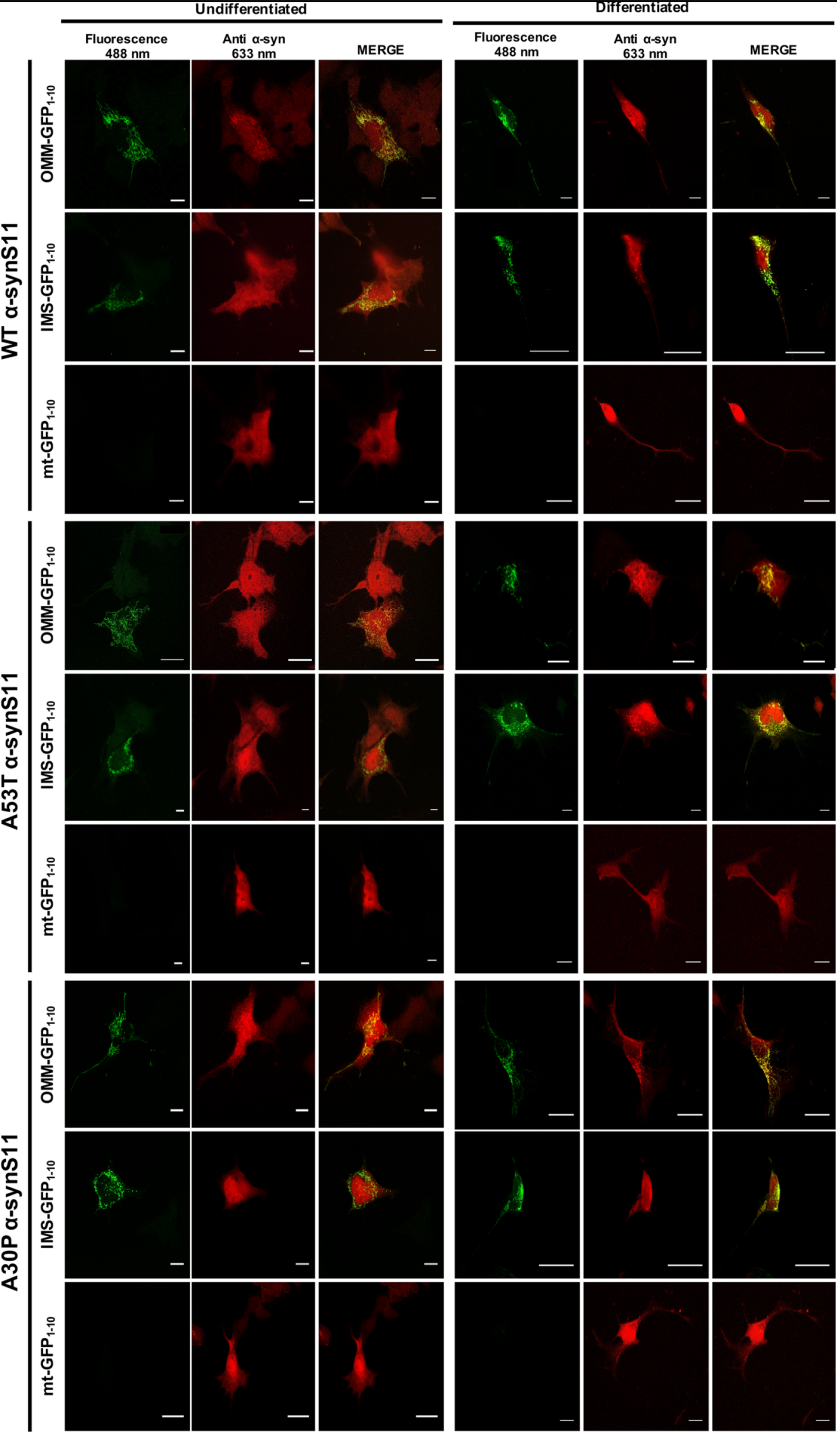
Sub-mitochondrial localization of wild type and mutant α-syn was analyzed in undifferentiated. **a** and differentiated **b** BE(2)-M17 dopaminergic-like cells by the co-expression of the OMM, IMS and mitochondrial matrix targeted GFP_1–10_ non-fluorescent moiety and of the WT, A53T and A30P α-synS11. Confocal images were acquired at 488 and 594 nm excitation wavelength. Transfected cells were incubated with an anti α-syn primary antibody and stained with an Alexa 633 conjugated secondary antibody. Complementation of the GFP probes was revealed by fluorescent acquisition at 488 nm excitation wavelength. Scale bar is 20 μm

**Fig. 3:**
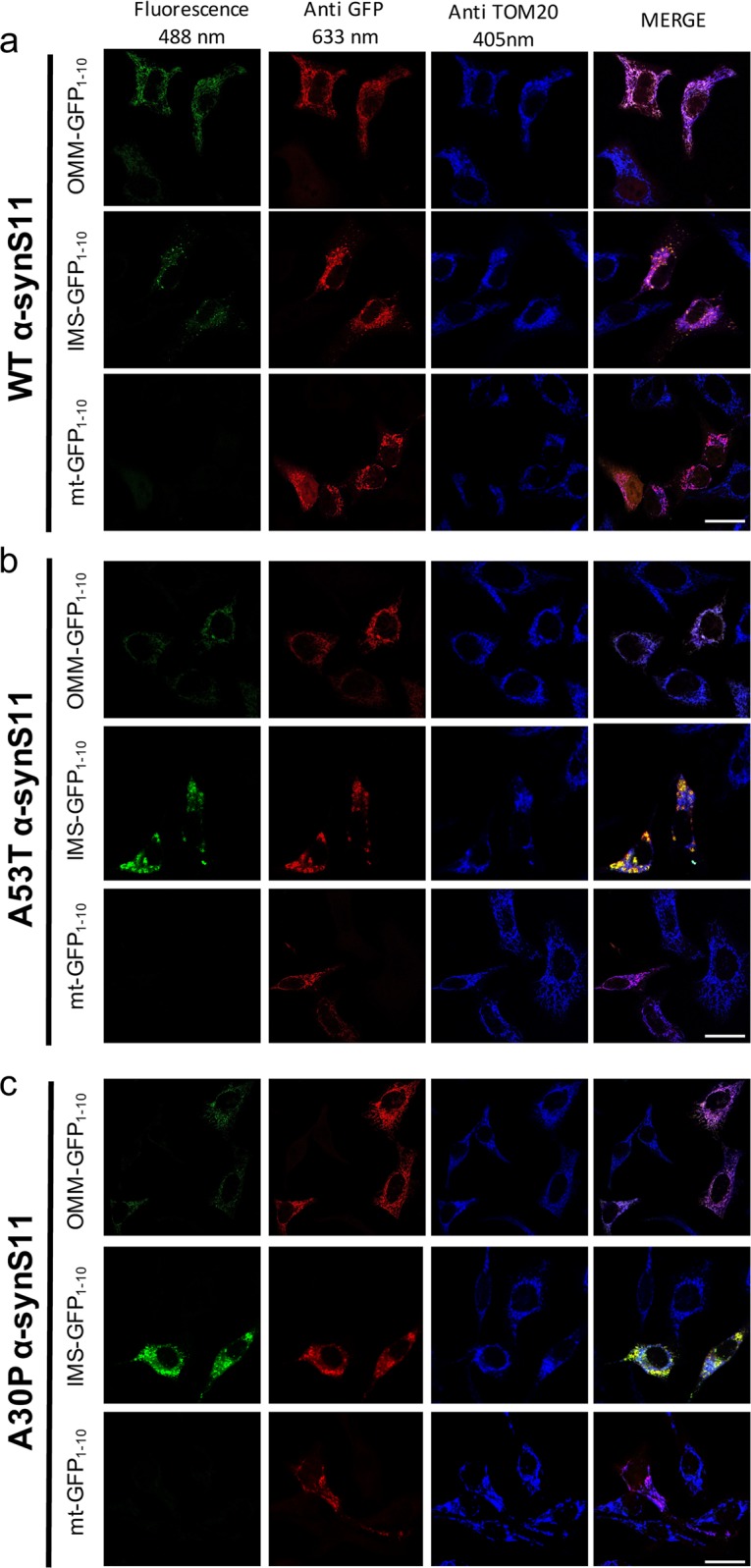
Expression of the OMM, IMS and mitochondrial matrix targeted GFP_1-10_ non-fluorescent moiety and of the. **a** WT α-synS11, **b** A53T α-synS11, and **c** A30P α-synS11. Complementation of the GFP probes was revealed by fluorescent acquisition upon excitation at 488 nm wavelength. GFP was detected using a GFP primary antibody while mitochondria were stained using an anti TOM20 antibody. Confocal images were acquired at 633 and 405 nm excitation wavelength. Cells were stained with anti GFP and anti TOM20 antibodies and observed at a confocal microscope at 405, 488 and 633 nm excitation wavelength. Scale bar is 20 μm

To investigate the sub-mitochondrial localization of WT α-syn and its pathologic mutants, we co-transfected HeLa (Fig. 1b) and SHSY5Y neuroblastoma (Fig. 1c) cells with the above described GFP_1-10_ constructs and the untargeted WT, A53T and A30P α-syn fused at their C-terminal with the S11 β-strand. The expression of α-syn was verified using an anti α-syn antibody, that has revealed a diffuse cytosolic pattern (Fig. 1b, c, in red). GFP complementation in cells overexpressing the OMM GFP_1-10_ and the IMS GFP_1-10_ with the untargeted WT, A53T and A30P α-synS11 revealed that a pool of overexpressed α-syn is localized both at the cytosolic surface of the outer mitochondrial membrane and in the intermembrane space (Fig. 1b, c, upper and middle panels). Interestingly, no fluorescence signal following excitation at 488 nm wavelength was detected in the mitochondrial matrix upon co-expression of the mtGFP_1–10_ and WT and mutant α-synS11, indicating that the overexpression of α-syn does not induce the accumulation of α-syn in the mitochondrial matrix (Fig. 1b, c lower panels). To confirm the above mentioned results in a different dopaminergic-like cell model, we also performed the experiments in undifferentiated and differentiated BE(2)-M17 cell lines (Fig. 2 left and right, respectively and Fig. S3) and, as in the case of HeLa and SHSY5Y cells, we detected α-syn at the OMM and the IMS but not in the mitochondrial matrix. The anti TOM20 immunostaining confirmed the mitochondrial specificity of GFP emission signals (Fig. 3a–c).

To support our findings obtained with splitGFP method and to assess whether α-syn variants differentially associate with the mitochondria, α-syn abundance in cytosolic and mitochondrial fractions were obtained from HeLa cells transfected with WT, A30P, or A53T α-syn expressing vectors was verified by Western Blot analysis (Fig. S4).

Quantification of mitochondrial α-syn amount in respect to mitochondrial content, calculated as the normalized mitochondrial α-syn/TOM20 ratio, showed no differences among the three different batches of transfected cells. Taken together, these results confirmed the presence of WT, A30P, or A53T α-syn in mitochondrial fraction but did not revealed any differences in their quantitative distribution.

To assess whether α-syn overexpression could affect mitochondrial bioenergetics, we analyzed mitochondrial and cytosolic ATP production upon cell stimulation with histamine, an inositol 1,4,5 tris-phosphate (InsP3)-producing agonist that mobilize Ca^2+^ from the endoplasmic reticulum. Mitochondrial (mtLUC) or cytosolic (cytLUC) recombinant luciferase probes and WT and mutant α-syn were co-expressed in HeLa cells and ATP levels were monitored upon addition of luciferin, as previously described^47^. Figure 1d shows the increment in light emission relative to the ATP production upon histamine stimulation, i.e., upon enhancement of energy requirement. Indeed, histamine stimulation by inducing transient mitochondrial Ca^2+^ concentration increases, stimulates Krebs cycle enzymes and enhances ATP production^47^. Mitochondrial ATP synthesis in cells overexpressing both the WT α-syn as well as the pathologic α-syn mutants is reduced of about 20% compared to control cells (whose levels are reported as 100%), suggesting that α-syn overexpression impaired ATP production independently from its PD-related mutations (mtLuc 100 ± 0.77 *n* = 14; WT α-syn 88.94 ± 1.02 *n* = 14; A53T α-syn 82.25 ± 0.59 *n* = 9; A30P α-syn 82,82 ± 0.51 *n* = 9). Cytosolic ATP levels were instead essentially unaffected by the presence of overexpressed α-syn (cytLuc 100 ± 1.95 *n* = 10; WT α-syn 95.66 ± 1.43 *n* = 11; A53T α-syn 96.65 ± 1.02 *n* = 14; A30P α-syn 96.10 ± 0.64 *n* = 14) (Fig. 1e).

### α-syn mitochondrial distribution is altered by the occurrence of pathogenic mutations and cellular stress conditions

**Fig. 4:**
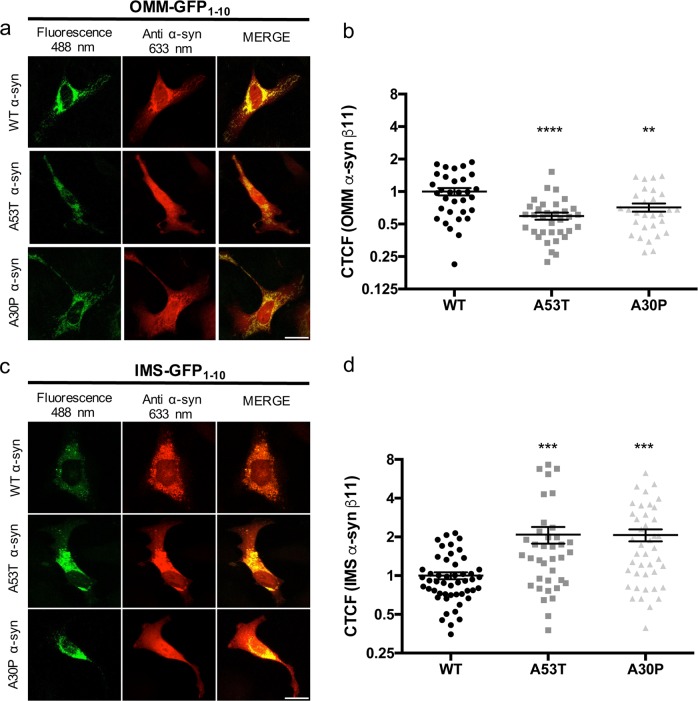
Quantification of wild type and mutant α-syn distribution at the OMM and IMS. Expression of the OMM. **a** and IMS **c** targeted GFP_1-10_ non-fluorescent moiety and of the WT, A53T and A30P α-synS11. Transfected cells were incubated with an anti α-syn primary antibody and stained with an Alexa 633 conjugated secondary antibody. Complementation of the GFP probes was revealed by fluorescent acquisition at 488 nm excitation wavelength **a**, **c**. The corrected total cell fluorescence (CTCF) was quantified as described in the ‘Materials and Methods’ section and reported as normalized values with respect to WT α-syn **b**, **d** (****p < 0.0001, ***p < 0.001, **p < 0.01). One-way ANOVA retrieved a *p* value of 0.0001 for **b** and **d**. Dunnett’s multiple comparison test retrieved a statistically significant difference between WT and A53T *p* < 0.001 and WT and A30P *p* < 0.01 **b** and between WT and A53T *p* < 0.001 and WT and A30P *p* < 0.001. Quantification of CTCF has been done on at least 29 cells from at least three independent experiments for each transfection condition. Scale bar is 20 μm

In order to investigate whether WT and mutant α-syn possess different propensity to localize at the mitochondria compartment, we quantified GFP fluorescence in HeLa cells co-transfected with OMM and IMS GFP_1–10_ along with WT, A53T and A30P α-synS11. From the images shown in Fig. 4a, it is evident that GFP fluorescent signal reconstituted by WT α-synS11 at the OMM is much more intense with respect to that reconstituted upon the overexpression of the pathogenic mutants. The quantification of the corrected total cell fluorescence (CTCF) revealed that WT α-synS11 has major propensity to localize at the OMM compared to mutant α-synS11 (CTCF: WT α-syn 1 ± 0.08 *n* = 32; A53T α-syn 0.59 ± 0.05 *n* = 34; A30P α-syn 0.71 ± 0.06 *n* = 29) (Fig. 4b), whereas, on the opposite, a greater fluorescence signal is detectable at the IMS for the mutant constructs (Fig. 4c), indicating that the occurrence of pathogenic mutations is able to favor the translocation of the protein across the OMM in the IMS (CTCF: WT α-syn 1 ± 0.06 *n* = 50; A53T α-syn 2.08 ± 0.31 *n* = 37; A30P α-syn 2.07 ± 0.22 *n* = 42) (Fig. 4d). To verify that the expression of α-synS11 constructs was similar among the different batches of transfected cells and that the same correlation exist between the immunoreactive α-synuclein signal and the fluorescence signal of reconstituted GFP we have quantified the red and the green fluorescence along the entire volume of the cells positive for reconstituted GFP_1–10_ and plotted them. As expected, a positive relationship is present for WT, A53T and A30P α-synS11 either at the OMM and at the IMS. However, a poor positive linear correlation is observed (Fig. S5) which is equivalent in all the batches of transfected cells (except for the A53T α-synS11 at the OMM, see the *R*^2^ values for comparison), suggesting that cell specific differences in the expression level of α-synS11 do not affect the observed differences in fluorescence intensities among WT, A53T and A30P α-synS11 within the same sub-mitochondrial compartment.

**Fig. 5:**
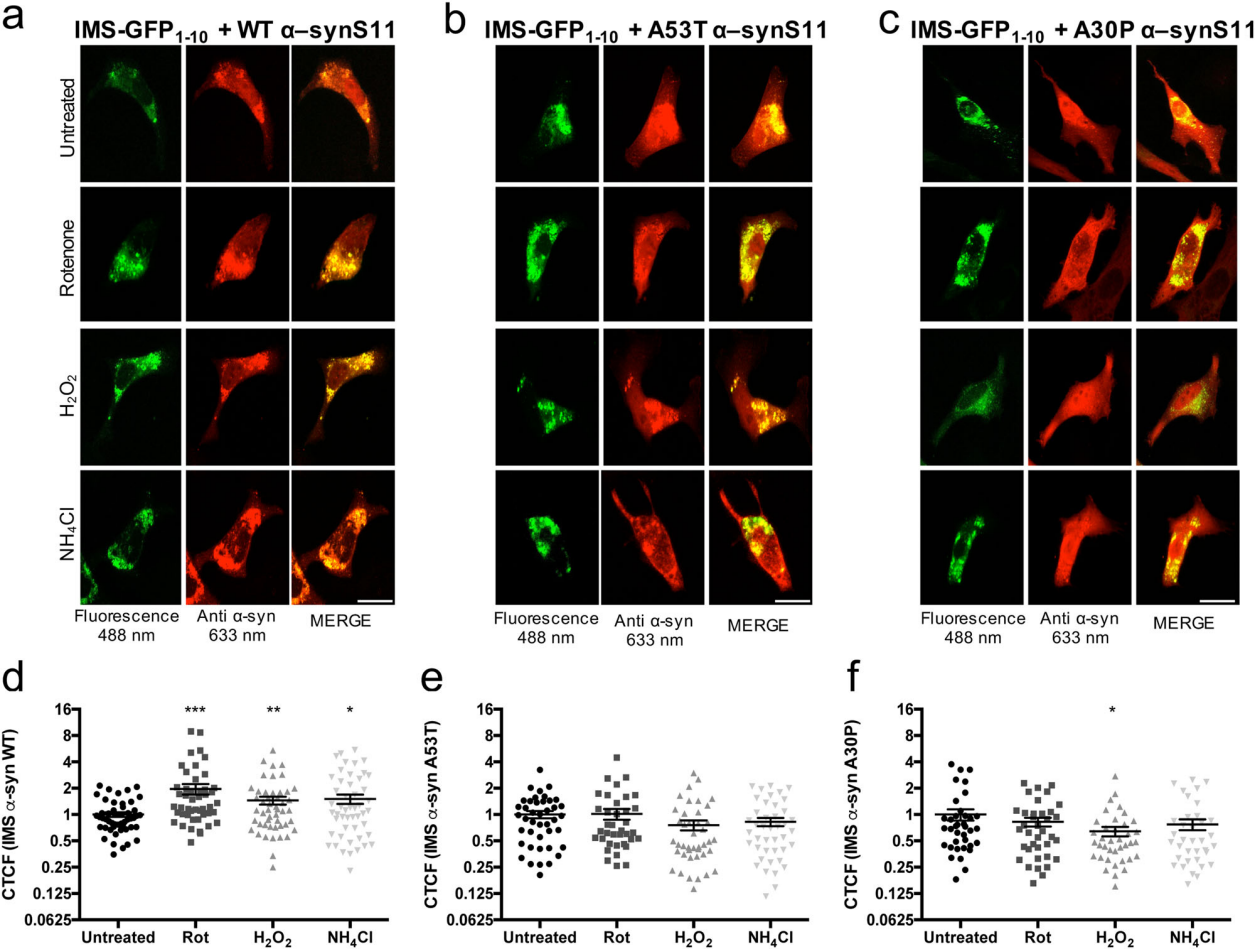
α-syn distribution at the IMS upon inhibition of complex I inhibitor or oxidative stress or inhibition of endosome-lysosome system acidification. HeLa cells were co-transfected with wild type **a** and mutants **b**, **c** α-synS11 and IMS GFP_1–10_, and treated with 10 μM rotenone for 12 h, 250 μM H_2_O_2_ for 12 h or 50 mM NH_4_Cl for 2 h at 37 °C in a 5% CO_2_ atmosphere, stained with an anti α-syn antibody and observed at a confocal microscope at 488 and 633 nm excitation wavelength. **d**–**f** CTCF was quantified as described in the ‘Materials and Methods’ section and reported as normalized values with respect to WT α-syn. (**d**, Student’s *t*-test: ***p < 0.001, **p < 0.01, *p < 0.05; One-way ANOVA *p*=0.0037. Dunnett’s multiple comparison test retrieved a statistically significant difference only for the Rot treated group, ***p < 0.001; **f** Student’s *t*-test: *p < 0.05; One-way ANOVA and Dunnett’s multiple comparison test retrieved no statistically significant differences between the groups). Quantification of CTCF has been done on at least 36 cells from at least three independent experiments for each condition. Scale bar is 20 μm

At the light of these results, we wanted to find specific cell conditions that may change α-syn distribution at the OMM or the IMS. We decided to evaluate this aspect by monitoring α-syn at the IMS. HeLa cells were transfected with IMS GFP_1–10_ and WT or mutant α-synS11 and treated with 10 μM rotenone for 12 h, 250 μM H_2_O_2_ for 12 h, or 50 mM NH_4_Cl for 2 h at 37 °C in a 5% CO_2_ atmosphere. As shown in Fig. 5a, d, there was a significant increase in WT α-syn in the IMS in treated HeLa cells compared to control (WT α-syn: control 1 ± 0.06 *n* = 50; rotenone 1.96 ± 0.27 *n* = 48; H_2_O_2_ 1.45 ± 0.15 *n* = 49; NH_4_Cl 1.51 ± 0.18 *n* = 54). Interestingly, upon the same stimuli, the IMS pool of α-synS11 mutants did not changed (Fig. 5b, c), as better appreciable from the quantitative analysis shown in Fig. 5e, f (for A53T α-syn: control 1 ± 0.10 *n* = 42; rotenone 1.02 ± 0.14 *n* = 37; H_2_O_2_ 0.76 ± 0.10 *n* = 42; NH_4_Cl 0.82 ± 0.09 *n* = 42 and for A30P α-syn: control 1 ± 0.15 *n* = 37; rotenone 0.82 ± 0.10 *n* = 37; H_2_O_2_ 0.64 ± 0.08 *n* = 38; NH_4_Cl 0.77 ± 0.11 *n* = 36).

At this point we were interested to assess whether the same conditions that favoured WT α-syn accumulation into IMS, may also lead to α-syn translocation into the mitochondrial matrix. Thus, we performed similar experiments using the mt GFP_1–10_ moiety: no fluorescence signal was detected for any of the α-synS11 constructs upon cells incubation with rotenone, H_2_O_2_ or NH_4_Cl (data not shown), suggesting that these treatments, in our experimental conditions, do not induce WT and mutant α-syn translocation into the mitochondrial matrix.

All together, these data suggest that the pathogenic mutations lead to α-syn accumulation in the IMS and that pharmacological treatments that impair complex I activity or increase oxidative stress or inhibit the endosome-lysosome system acidification mimic the same effect for WT α-syn.

### The artificial targeting of WT α-syn, but not of its pathogenic mutants, to the mitochondrial matrix sustains ATP synthesis in a Complex III-dependent manner

Although we were not able to detect the presence of α-syn in the mitochondrial matrix (neither under basal nor under stress conditions), recent findings strongly supported the view that α-syn can indeed reach the mitochondrial matrix and modulate ATP production and/or mitochondrial membrane potential^33–35,38,48^, indicating that in other cell types or under specific experimental conditions α-syn could translocate in the mitochondrial matrix.

**Fig. 6:**
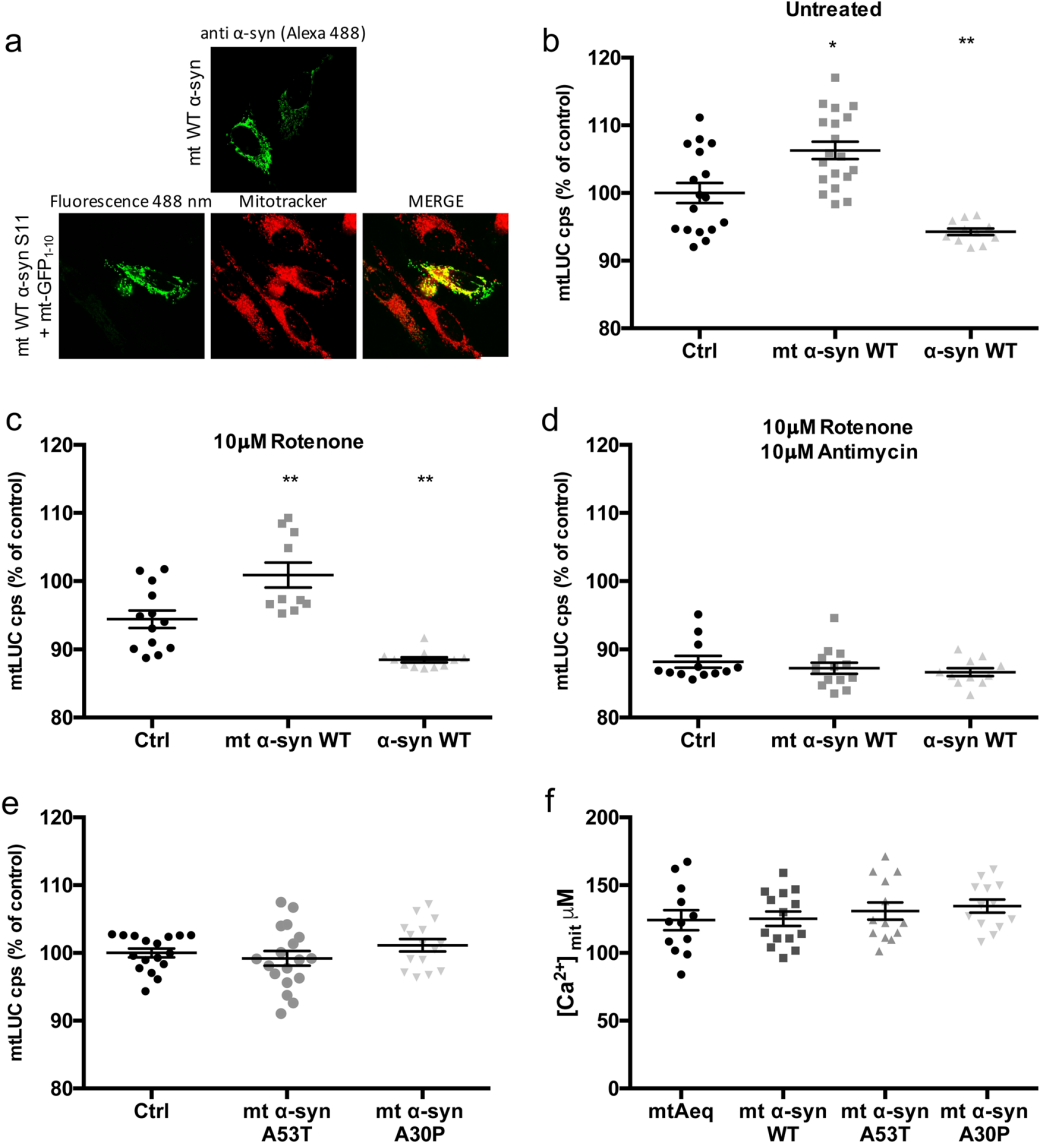
WT and mutant α-syn targeting to the mitochondrial matrix differently affects mitochondrial ATP levels. **a** Complementation of the mitochondrial matrix targeted GFP_1-10_ non-fluorescent moiety upon co-expression of the targeted mt WT α-synS11. The mitochondrial localization was verified by mitotracker staining. Confocal images were acquired at 488 and 594 nm excitation wavelength. **b**, **c**, **d** Histograms showing mitochondrial ATP production upon histamine stimulation measured by mtLuc probe in HeLa cells overexpressing untargeted or mitochondrial matrix targeted α-syn in HeLa cells untreated **b** or treated with 10 μM rotenone **c** or **d** 10 μM rotenone + 10 μM antimycin for 12 h at 37 °C in a 5% CO_2_ atmosphere. **e** Histograms showing mitochondrial ATP production upon histamine stimulation measured by mtLuc probe in HeLa cells overexpressing mitochondrial matrix targeted mt WT, mt A53T and mt A30P α-syn. Average values are shown as mean % calculated from the counts per second (cps) with respect to control cells. **f** Mitochondrial Ca^2+^ transients upon stimulation with 100 μM histamine were measured in HeLa cells by co-transfecting mtAEQ and empty vector, or mt WT, mt A53T and mt A30P α-syn. Bars represent mean Ca^2+^ peak values upon histamine stimulation. Data were collected from at least 10 coverslips/conditions from three independent experiments. (One-way ANOVA coupled with Dunnett’s multiple comparison test retrieved a statistically significant difference between the groups: **p < 0.01, *p < 0.05). At least 10 independent measurements for three independent transfections have been done for each construct. Scale bar is 20 μm

Indeed, it has been shown that exogenous monomeric WT α-syn, but not its A30P mutant, is able to physically interact with the α subunit of the ATP synthase and to increase its activity in primary neuron/glia co-cultures from mice cerebral cortex^38^. Thus, we decided to directly investigate possible action on mitochondrial metabolism of α-syn artificially targeted to the mitochondrial matrix. To this end, we generated a mitochondrial matrix-targeted α-synS11 (mtα-synS11) by adding the same targeting sequence that we have fused to the GFP_1-10_ fragment. mt α-synS11 efficiently complemented mtGFP_1-10_, as revealed by the mitochondrially localized green fluorescent signal observed following excitation at 488 nm wavelength (Fig. 6a). To evaluate its possible impact on mitochondria function, mitochondrial ATP production upon cells stimulation with histamine was measured in parallel both in cells overexpressing mt α-synS11 or untargeted α-synS11. As shown in Fig. 6b, while the overexpression of untargeted α-syn reduced mitochondrial ATP synthesis (as already shown in Fig. 1e), the mt α-synS11 was able to enhance ATP production (Mean values (% cps): mt Luc 100 ± 1.49 *n* = 17; mt α-syn 106.29 ± 1.28 *n* = 19; α-syn 94.27 ± 0.50 *n* = 11). Interestingly, mt α-synS11 still exerted this effect in the presence of complex I inhibitor rotenone (Mean values (% cps): mt Luc 94.45 ± 1.29 *n* = 13; mt α-syn 100.88 ± 1.83 *n* = 10; α-syn 88.48 ± 0.39 *n* = 11) (Fig. 6c), while the ability to promote ATP synthesis was lost in the presence of the complex III inhibitor antimycin, (Mean values (%cps): mt Luc 88.20 ± 0.86 *n* = 12; mt α-syn 87.24 ± 0.83 *n* = 13; α-syn 86.66 ± 0.58 *n* = 11) (Fig. 5d). Of notice, pathogenic mutants of α-syn artificially targeted to the mitochondrial matrix are not able to increase mitochondrial ATP synthesis upon cell stimulation, (Mean values (cps): mt Luc 100 ± 0.63 *n* = 17; mt A53T α-syn 99.20 ± 1.08 *n* = 18; mt A30P α-syn 101.13 ± 0.90 *n* = 15) (Fig. 6e).

To check whether the observed increases in ATP production could be related to possible effects of mt α-synS11 on mitochondrial Ca^2+^ uptake, as previously shown for untargeted cytosolic α-syn^32^, mitochondrial Ca^2+^ measurements were performed using mitochondrially targeted photoprotein aequorin^49,50^. No alterations in mitochondrial Ca^2+^ levels were registered upon histamine stimulation in cells overexpressing the wt or the mutated mitochondrial matrix targeted α-syn constructs, indicating that the sustained ATP levels are not driven by an increase in mitochondrial Ca^2+^ transients that could stimulate the Krebs cycle (Peak values (μM): mtAEQ 124.19 ± 7.40 *n* = 12; mt WT α-syn 125.18 ± 5.34 *n* = 14; mt A53T α-syn 130.98 ± 6.35 *n* = 13; mt A30P α-syn 134.57 ± 4.77 *n* = 13) (Fig. 6f). These findings suggest that WT α-syn, resident in the mitochondria matrix, could play a direct role on ATP production by the modulation of the respiratory chain (possibly through complex III activity) and that the presence of pathogenic mutations compromises this function.

### VDAC 1-3 are not responsible for α-syn translocation inside mitochondria

**Fig. 7:**
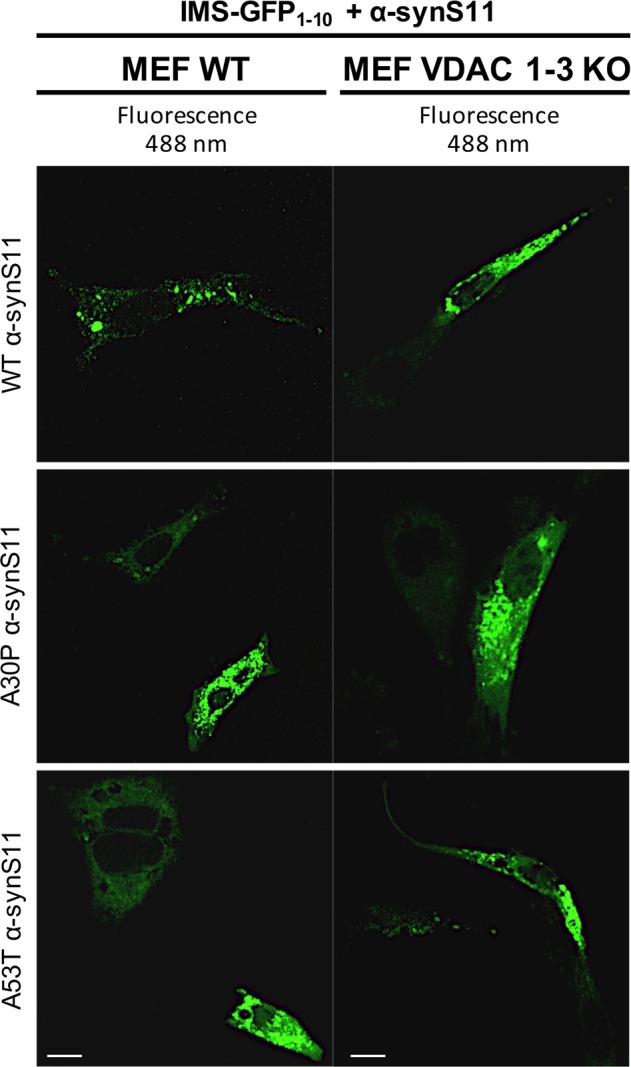
α-syn mitochondrial import is not affected by VDAC 1-3 knock out in MEF cells. The IMS targeted GFP_1-10_ non-fluorescent moiety and of the WT, A53T and A30P α-synS11 were expressed in WT and VDAC 1-3 KO MEF. Complementation of the GFP probes was revealed by fluorescent acquisition at 488 nm excitation wavelength. Scale bar is 20 μm

The evidence that a portion of α-syn resides at the IMS, requires the direct translocation of the protein from the cytosol across the outer mitochondrial membrane. Recent in vitro experiments using the Voltage Anion Channel (VDAC) reconstituted into planar lipid membranes suggested that α-syn is able to interact with/translocate through the VDAC1 to reach the IMS^51,52^. To verify the possibility that VDAC could represent a docking site for α-syn entry in IMS in living cells, we transfected mouse embryonic fibroblasts (MEF) WT and MEF VDAC 1-3 KO with IMS GFP_1–10_ and WT or mutant α-synS11: we could expect that the ablation of the channel could prevent α-syn translocation across the OMM. Surprisingly, we have found that VDAC 1–3 absence does not alter the ability of α-syn (WT and mutants) to translocate to the IMS, as revealed by the specific complementation of the fluorescent probe and the resulting green fluorescence emission (Fig. 7). These data clearly suggest that VDAC 1–3 are not essential for α-syn translocation inside mitochondria in intact cells. Whether this indicate that VDAC 1–3 proteins are not the only channels implicated in α-syn translocation or that α-syn takes completely different routes to reach the IMS (i.e., by TOM40 as previously suggested^14^) remains to be further elucidated.

### In vivo localization of α-syn at the OMM and the IMS, but not the mitochondrial matrix

**Fig. 8:**
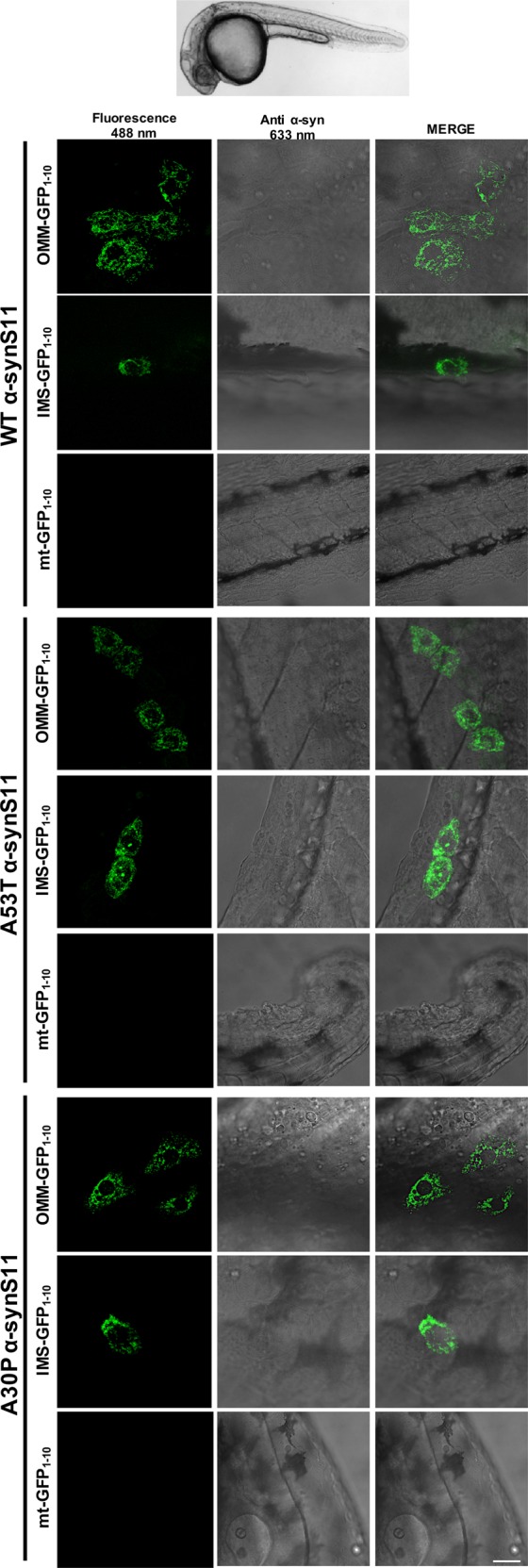
Live imaging of SPLIT GFP reconstitution in 1 dpf zebrafish embryos. Wild type zebrafish embryos at 1-cell stage were injected with a plasmid expressing human WT or A53T and A30P mutants αsynS11 together with a plasmid expressing OMM-GFP_1–10_, IMS-GFP_1–10_ or mt-GFP_1–10_. For the injections, the concentration of each plasmid was 50 ng/ul. After 1 day, images were collected. Each picture is the merge of several planes. Scale bar is 20 μm

The above mentioned experiments suggested the existence of a mitochondrial pool of α-syn at the OMM and at the IMS while, under basal conditions we could never detect it within the mitochondrial matrix. To further validate these findings in a more complex system, we decided to test for the first time the possibility to follow the translocation of a protein of interest within mitochondrial sub compartments with our splitGFP system in the living vertebrate *Danio Rerio*. To this aim, fertilized oocytes at the stage of 1 cell have been microinjected with the plasmids encoding for α-synS11 (either WT, A53T or A30P) together with the OMM GFP_1–10_, the IMS GFP_1-10_ or the mtGFP_1–10_ at the final concentration of 100ng/μl. Under these conditions, a mosaic expression is expected to take place. Injected embryos at 1dpf have been dechorionated, anesthetized and mounted in low melting agarose. Confocal z-stacks were acquired in vivo and, as shown in Fig. 8, a clear and strong signal can be detected with the OMM and the IMS GFP1-10 probes co-injected with either WT, A53T or A30P α-synS11, suggesting that a fraction of α-syn is indeed present at the OMM as well as the IMS in living zebrafish. On the other side, no mitochondrial signal could be detected by using the matrix targeted GFP_1–10_ supporting the idea that, under basal conditions, α-syn does not reach the mitochondrial matrix. These results have confirmed the findings we have obtained in HeLa and dopaminergic cells and, intriguingly, have also demonstrated that our splitGFP assay is suitable for in vivo settings.

## Discussion

Mitochondrial dysfunctions and α-syn accumulation into Lewy bodies are both considered as key events in the progressive loss of dopaminergic neurons leading to PD manifestation. Although the precise role of α-syn in the neurodegenerative process is largely unknown, several lines of evidence have highlighted its possible involvement in the regulation of mitochondrial functions. For these reasons, numerous studies have focused their attention on α-syn contribution to the control of mitochondrial activities^53,54^. Nevertheless, whether mitochondrial dysfunctions represent a secondary event in α-syn-induced alterations or are directly triggered by α-syn at the mitochondrial level is still unclear. Moreover, the absence of techniques that allow to specifically monitor the protein’s distribution inside organelles, further intricate the road to a complete understanding of α-syn physio/pathology inside mitochondria. Here, we employed a split-GFP based bimolecular fluorescence complementation (BiFC) tool^40,42^ developed by our group to selectively monitor DJ-1 sub-mitochondrial distribution^43^ and further improved with a novel non fluorescent GFP_1–10_ moiety targeted to the IMS^55^ to selectively follow proteins translocation inside the inter membrane space. Our analyses revealed that a fraction of WT, A30P and A53T α-syn is localized at mitochondria both at the OMM and in the IMS, and that the overexpression of α-syn resulted in a decrease in mitochondrial ATP production upon cell stimulation, as reported by others^19^. This result was partially unexpected considering that we had previously shown that α-syn overexpression was able to enhance ER-mitochondria Ca^2+^ transfer^32^, thus leading to the possibility that increased mitochondrial Ca^2+^ transients could augment ATP production. But it could be rationalized assuming that increased ER-mitochondria tethering may represent a compensatory response to cope with reduced mitochondrial ATP production in the presence of increased amount of α-syn and that in the absence of this compensatory response the deficit in ATP synthesis could be even greater. Interestingly, while the WT protein is prevalently localized at the OMM, the pathologic mutants show a major IMS distribution, in agreement with previous results showing a lower degree of localization of both mutant α-syn species to the endoplasmic reticulum-mitochondria interface compare with WT and a concomitantly higher degree of localization to the pure mitochondrial fraction^18^. Interestingly enough, we were able for the first time to test this system in a living vertebrate, i.e., the zebrafish *Danio Rerio* and confirm the presence of α-syn at the OMM and at the IMS, as well as its absence in the mitochondrial matrix under basal conditions. Our data indicate that conditions of cellular stresses promote the translocation of the WT protein to IMS, but not that of the mutant species, that are more abundant per se in this compartment, suggesting the possibility that the recruitment of α-syn from the OMM surface inside mitochondria, i.e., in the IMS and, possibly into the matrix where it enhances ATP production, could represent an initial step to counteract cell impairment and sustain bioenergetics. However, whenever α-syn accumulation become excessive, α-syn may fail to be imported in the mitochondrial matrix and stacked at the IMS where could be responsible for mitochondrial fragmentation, a mechanism of action previously suggested^29,30^ and recently confirmed^56^ to occur. Whether the different behavior of WT and mutant α-syn reflects possible role of α-syn inside mitochondria, i.e., in controlling mitochondrial dynamics^54^ or ATP production^38^, or is driven by a specific import of misfolded/aggregation prone proteins into mitochondria for their degradations, as recently suggested^57^, deserves further investigation. Interestingly, Pozo Devoto and coworkers have recently shown that α-syn plays a direct physiological role in mitochondrial transport and morphology and that due to the different affinity for membrane lipids the association of WT and A53T or A30P α-syn with the mitochondria was different, thus suggesting that their effects on mitochondria are also dose-dependent^56^.

Since recent evidence have shown that in primary neuron/glia co-cultures exogenous monomeric α-syn is able to physically interact with the α subunit of the ATP synthase^38^, indicating a distinctive localization of the protein inside the mitochondrial matrix, we decided to evaluate phenotypes associated with this peculiar α-syn distribution by artificial targeting α-syn protein to the mitochondrial matrix. Interestingly, and in agreement with previous studies^38^, mitochondrial matrix-targeted WT α-syn is able to sustain mitochondrial ATP synthesis, while mutant species targeted to the mitochondrial matrix do not promote mitochondrial ATP production. We have found that this action is dependent on complex III activity, since it is prevented by the incubation with the complex III inhibitor antimycin. In this context, one may speculate that, in neurons, a pool of α-syn exerts a physiological role inside the mitochondrial matrix where it is able to increase ATP synthase activity ensuring mitochondrial health and synaptic functions and that another pool of α-syn may be recruited to this site upon stress condition to further sustain ATP production through the modulation of complex III activity. The occurrence of mutations may lead to a loss of function that causes energy depletion and neuronal cell toxicity, thus initiating the degenerative process in PD.

## Materials and methods

### DNA constructs

The full length α-syn WT, A53T and A30P -**β**11 construct have been generated by PCR using the following primers: α-syn-S11 (HindIII) For 5′- GTTCAAGCTTATGGATGTATTCATGAAAGG -3′; α-syn-S11 (XhoI) Rev. 5′-ACTTCTCACTCGAGTTATGTGATGCCGGCGGCGTTCACGTA CTCGTGCAGCACCATGTGGTCCCGGCTGCCGCCGCCGCTGCCGCCGTCGCCGGCTTCAGGTTCGTAGTCTTG-3′ The DNA constructs encoding for the human α-syn WT, A30P and A53T used as a template are a kind gift of Prof. Alessandro Negro (Department of Biomedical Sciences, University of Padova). The C-terminus **β**11-tagged Cytochrome-c was produced by DNA synthesis (Thermo Scientific). Untargeted, mitochondrial matrix- and OMM-targeted humanized GFP 1–10 expressing vectors were generated by PCR amplification from the GPI-GFP1-10 template using forward primers containing the mitochondrial matrix presequence of the subunit VIII of cytochrome c oxidase and the N-terminal 33 amino acids sequence of TOM20 protein^43^.

The GFP_1**–**10_ targeted to the intermembrane space (IMS) has been created by genetic fusion to the leader sequence of the IMS protein LACTB^44^ and created by DNA synthesis (Thermo Scientific).

### Cell cultures and transfection

HeLa, SHSY5Y cells and VDAC1/3^−/−^ MEFs were grown in DMEM high glucose (Euroclone) containing 10% fetal bovine serum (FBS, Gibco), supplemented with 100 U/ml penicillin (Euroclone) and 100 μg/ml streptomycin (Euroclone), in a humidified atmosphere containing 5% CO_2_. Undifferentiated BE(2)-M17 cells were maintained in a 1:1 mixture of Ham's F12 and Dulbecco’s Modified Eagle’s Medium (Gibco) supplemented with 10% fetal bovine serum and grown in a humidified incubator at 37 °C in the presence of 5% CO_2_. The cell medium was replaced every 2 days, and the cells were sub-cultured once confluence was reached. In all of the experiments, the cells were used at early passages (P1-5 after purchase). Differences in morphology between proliferative and differentiated cells were evaluated by phase contrast light microscopy.

Cells were seeded onto 13 or 24 mm diameter glass coverslips and transfection was performed at 60–80% confluence using Lipofectamine TM 2000 Transfection Reagent (Life Technologies) for SHSY5Y, BE(2)-M17 and MEFs and the calcium-phosphate procedure for HeLa cells. To analyze the presence of α-syn in the mitochondrial sub-compartments after differentiation, BE(2)-M17 cells were seeded onto coverslips pre-coated with poly-D-lysine^58^. After 24 h, differentiation was induced by the addition of retinoic acid (RA) at concentrations of 5 μM. Fresh media containing RA was provided every 2 day and 7 days later the cells were transfected with the vectors containing the coding sequence for targeted GFP_1–10_ chimerae and WT, A53T or A30P α-syn. After 48h the samples were fixed and processed for immunocytochemistry. For Ca^2+^ measurement cells were co-transfected with aequorin construct targeted to the mitochondrial matrix^49^. Cells were generally analyzed 24–48 h after transfection.

### Immunocytochemistry analysis

After 48 h of transfection, cells plated on 13 mm glass coverslips were washed twice with phosphate-buffered saline (PBS: 140 mM NaCl, 2 mM KCl, 1.5 mM KH_2_PO_4_, 8 mM Na_2_HPO_4_ pH 7.4) and fixed for 20 min with 3.7% formaldehyde in PBS. Cells permeabilization was performed by 20 min’ incubation with 0.1% Triton X-100 in PBS, followed by 30 min’ wash with 1% gelatin (type IV, from calf skin, Sigma) in PBS. The coverslips were then incubated for 90 min at 37 °C in a wet chamber with the specific primary antibody diluted in PBS: anti-GFP (Santa Cruz, sc-9996) anti-alpha synuclein (Santa Cruz, sc-12767), anti-TOM20 (Santa Cruz, sc-11415). Staining was revealed by the incubation with specific AlexaFluor 405, 488 or 633 secondary antibodies, 1:50 (Life technologies) for 45 min at room temperature. Fluorescence was detected with a Leica SP5 confocal microscope and analyzed by ImageJ software.

### Quantification of BiFC at the OMM and IMS

To quantify fluorescence signals, the corrected total cell fluorescence (CTCF) was calculated according to the protocol described in^59^. Briefly, a complete z-stack of cells showing a clear fluorescence signal was acquired using a Leica SP5 confocal microscope. The total corrected cell fluorescence (CTCF) was calculated by selecting the cell using the freehand selection of Fiji in the drawing/selection polygon tool and the area, the integrated density and the mean grey value are measured while the remaining signal coming from outside the cell was removed. Area, integrated density and mean grey value were then measured within the cells and three selected non-fluorescent area in the image were chosen as background. At least three additional selections from a non-fluorescent region next to the cell of interest were acquired and considered as background. The CTCF was calculated as follow: integrated density – (area of selected cell×mean fluorescence of background readings). The calculated CTCF was then normalised against the CTCF values of wt α-syn-expressing cells. The results are shown as fold change increase/decrease over wt α-syn expressing cells CTCF levels.

### Luciferase assay

Luciferase luminescence was measured, as previously described^47^. HeLa cells co-transfected with a mitochondrial luciferase chimera (mtLuc^47^) were perfused at 37 °C with KRB (125 mM NaCl, 5 mM KCl, 1 mM Na_3_PO_4_, 1 mM MgSO_4_, 20 mM HEPES, pH 7.4, 37 °C) containing 1 mM CaCl_2_, 5.5 mM glucose and 20 μM luciferin. In total 100 μM histamine was added to the perfusion medium to induce mitochondrial ATP synthesis^47^. For each measurement, the light emission (cps, counts per second) after histamine application was normalized on cps emitted after luciferin addition. Where indicated, 10 μM rotenone and 10 μM antimycin were incubated for 12 h at 37 °C in a 5% CO_2_ atmosphere.

### Aequorin measurements

After 48 h of transfection mitochondrial low affinity aequorin (mtAEQ) was reconstituted by incubating cells for 1.5 h with 5 μM wt coelenterazine (Santacruz) in DMEM supplemented with 1% fetal bovine serum at 37 °C in a 5% CO_2_ atmosphere. After reconstitution, cells were transferred to the chamber of a purpose-built luminometer, and Ca^2+^ measurements were started in Krebs-Ringer modified buffer (KRB,125 mM NaCl, 5 mM KCl, 1 mM Na_3_PO_4_, 1 mM MgSO_4_, 5.5 mM glucose, 20 mM HEPES, pH 7.4, 37 °C) medium supplemented with 1 mM CaCl_2_ by stimulating HeLa cells with 100 μM histamine. The experiments were terminated by cell permeabilization with 100 μM digitonin in a hypotonic Ca^2+^-rich solution (10 mM CaCl_2_ in H_2_O) to discharge the remaining unused aequorin pool. The light signal was collected and calibrated off-line into Ca^2+^ concentration values using a computer algorithm based on the Ca^2+^ response curve of mitochondrial aequorin, as previously described^60^.

### Live imaging of αsyn sub mitochondrial localization in Zebrafish

The human WT, A53T or A30P mutant αsynS_11_ plasmids were injected into 1 cell stage WT eggs together with a plasmid expressing OMM-GFP_1–10_, IMS-GFP_1–10_ or mt-GFP_1–10_. For injections, all plasmids were diluted in Danieau solution (58 mM NaCl, 0.7 mM KCl, 0.4 mM MgSO4, 0.6 mM Ca (NO3)2, 5 mM HEPES pH 7.6) and 0.5% phenol red. For the injections, the concentration of each plasmid was 50 ng/ul. At 1dpf, about 30 embryos were screened for fluorescence, dechorionated and anesthetized. For in vivo imaging, about one third of the injected embryos showed fluorescent signal and half were anesthetised and mounted on 35 × 10 mm glass bottom Petri dishes (Ted Pella, INC. Prod. No. 14023-20) in low melting agarose (1.3%, Euro- Clone). Fish water containing tricaine methanesulfonate 0.61 mM (Sigma) was added in the Petri dishes, in order to keep fish anesthetized. Mounted fish were imaged at RT (20–23 °C) using a Leica TSC SP5 inverted confocal microscope, using a HCX PL APO ×63/numerical aperture 1.40–0.60. To image reconstituted GFP, a complete z-stack of the cell was acquired every 0.29 μm and shown as Z-projection of several planes.

### Statistical analysis

All of the data are representative of at least three independent experiments unless otherwise indicated. The sample was chosen by considering a power of 80%, a two side alpha error of 0.05 and a beta error of 0.2. Values are expressed as mean ± SEM. Significant differences are determined by one-way ANOVA with Dunnett’s multiple comparison test for multiparametric analysis of the different groups. Student’s unpaired two-tailed *t* test was used for two experimental comparisons. All statistical analyses were performed using GraphPad Prism version 6.00 for Mac OS X, GraphPad Software, La Jolla California USA. A *p* value ≤0.05 was considered statistically significant.

## Supplementary information

Supplementary material
