# Supplementary material for: A split-GFP tool reveals differences in the sub-mitochondrial distribution of wt and mutant alpha-synuclein
Source: Cell Death Dis. 2019 Nov 12;10(11):857. doi: 10.1038/s41419-019-2092-1 (PMC6851186; doi:10.1038/s41419-019-2092-1)
Supplement: Supplementary file 1 — Supplementary material [file 41419_2019_2092_MOESM1_ESM.pdf]

Supplementary Figure 1

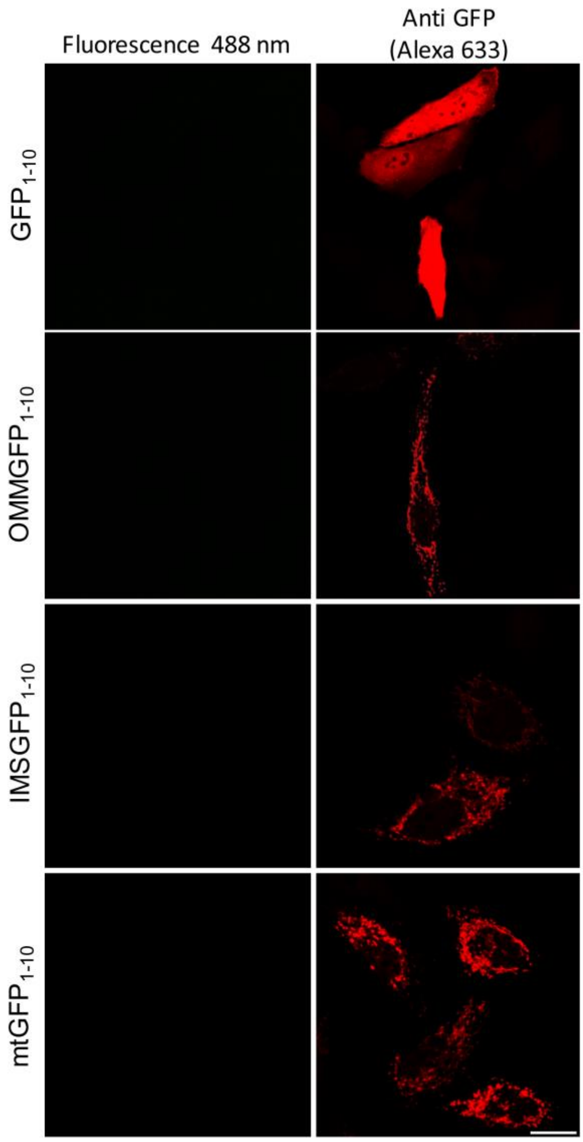

Figure S1. Immunofluorescence showing the localization and the non-fluorescent GFP_{1-10} fragment. HeLa cells were transfected with the indicated GFP_{1-10} probes, immunostained with an anti-GFP primary antibody and an Alexa 633 conjugated secondary antibody, images were acquired at 488 and 633 nm excitation wavelength. Scale bar is 20μm.

Supplementary Figure 2

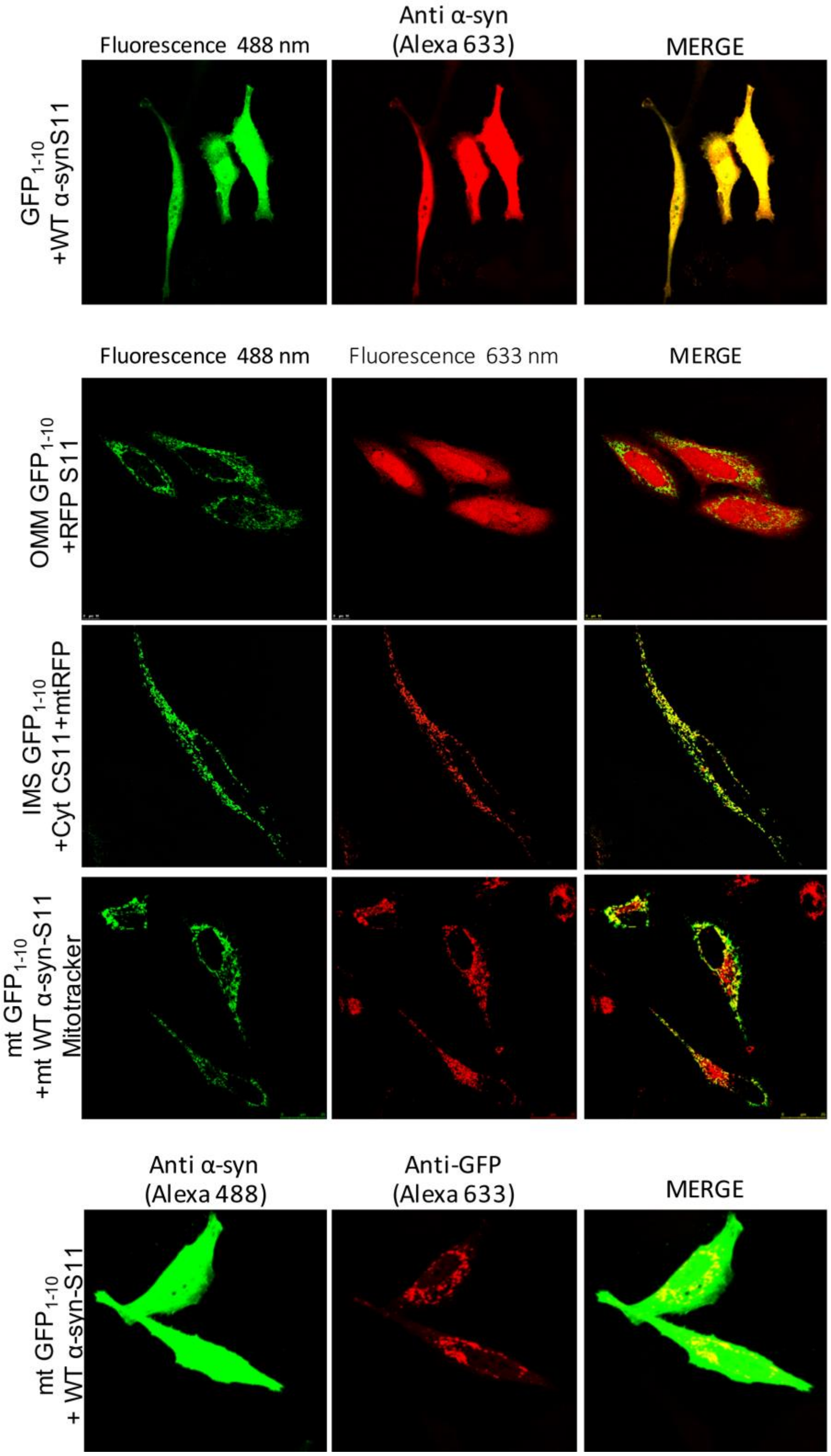

Figure S2. Immunofluorescence showing the localization and the self-complementation of the GFP_{1-10} probes and constructs used in this study. HeLa cells were co-transfected with the indicated GFP_{1-10} probes and S11 tagged constructs (RFPS11, cytosolic red fluorescent protein; CytCS11, Cytochrome c tagged with S11; mt WT α-syn, mitochondrially tagged WT α-syn; WT α-syn, cytosolic untagged α-syn), reconstitution occurs within the specific compartment. Cells are immunostained with an anti- α-syn or anti-GFP primary antibody and an Alexa 633 conjugated secondary antibody, images were acquired at 488 and 633 nm excitation wavelength.

Supplementary Figure 3

Undifferentiated BE(2)-M17 **Differentiated BE(2)-M17**

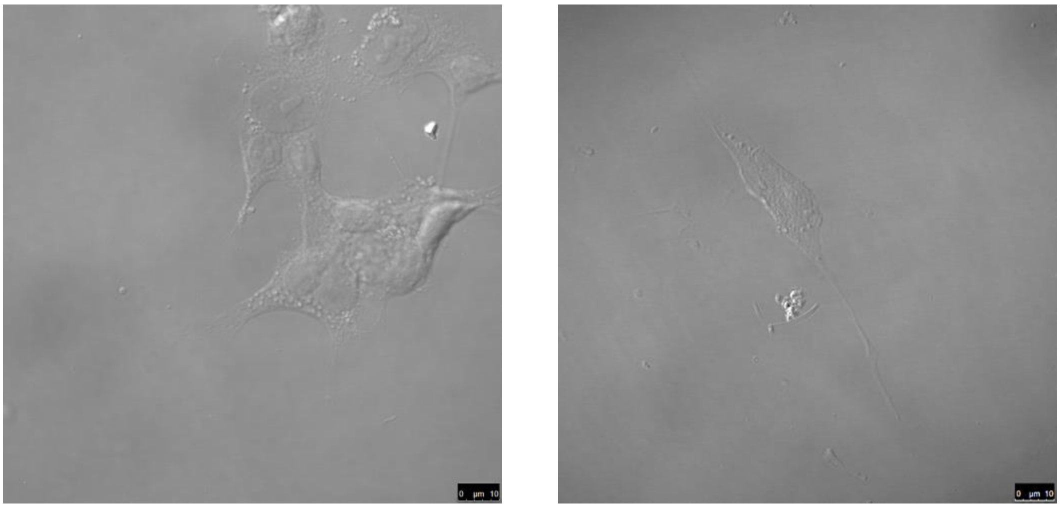

Figure S3. Bright Field images of undifferentiated (left) and differentiated BE(2)-M17 dopaminergic-like cells.

Supplementary Figure 4

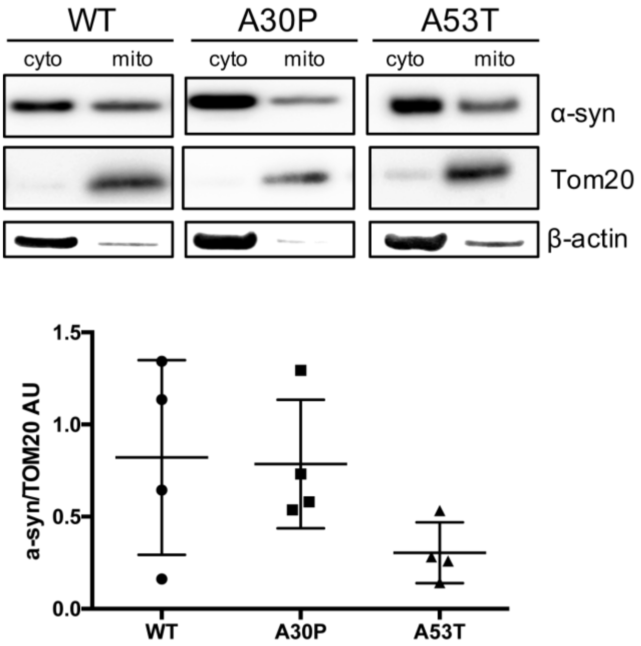

Figure S4. Western blotting analysis (top panel) and quantification (bottom panel) of subcellular fractionation of HeLa cells overexpressing the indicated S11-tagged α-syn constructs. Cell fractionation has been performed as reported in [44, 45]. One-way ANOVA analysis retrieved no statistically significant differences. The data are representative of four independent experiments.

Supplementary Figure 5

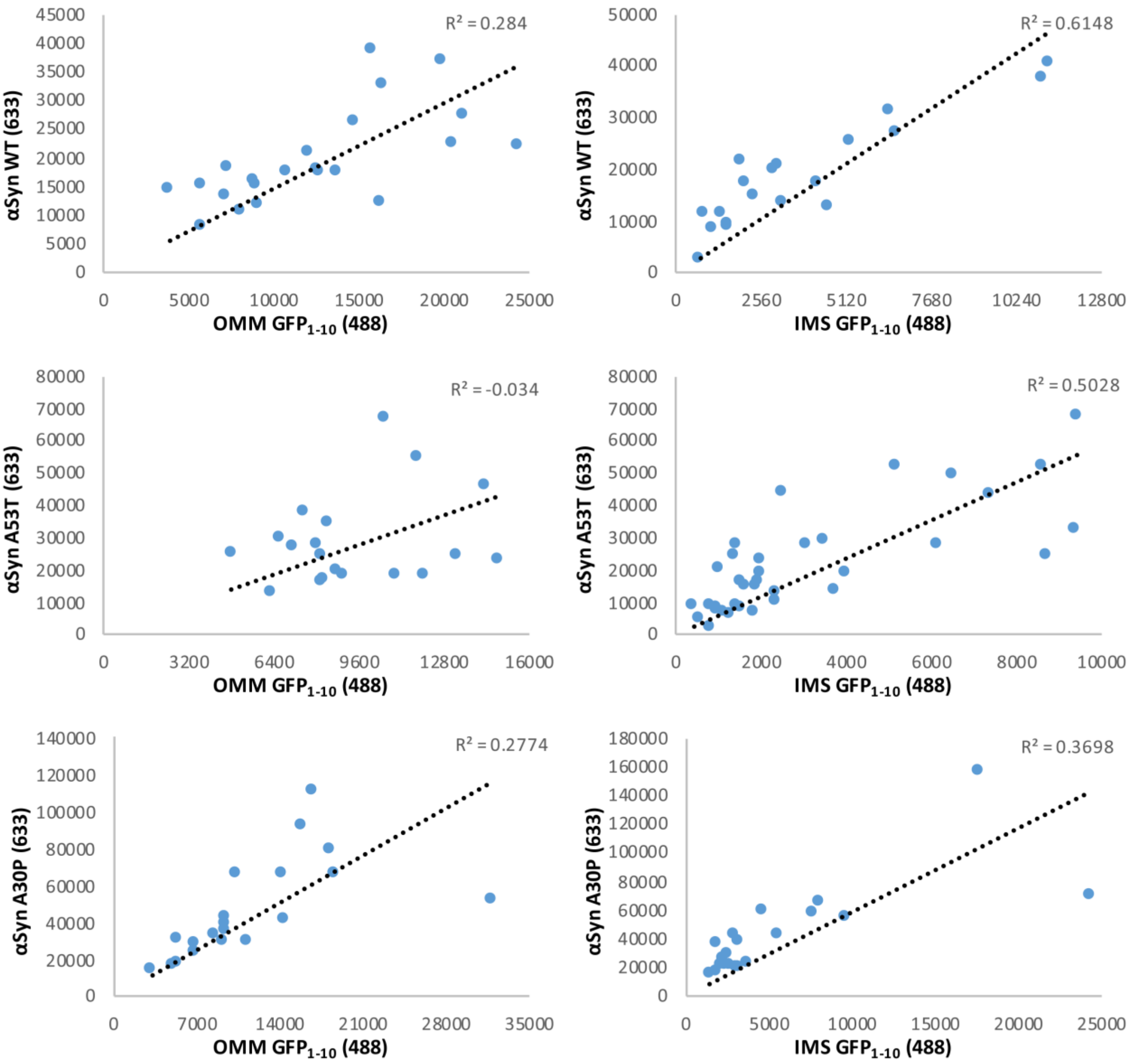

Figure S5. Correlation between the immunofluorescence intensity of α-syn WT, A53T and A30P and the fluorescence intensity of reconstituted GFP at the OMM (Left) and at the IMS (Right). Fluorescence intensity has been quantified by Image J analysis. The correlation coefficient is shown. The data are representative of at least two independent experiments and at least 19 cells were tested for each transfection condition.
